# Two Atypical Clinical Cases of a Rare Lymphoma: Peripheral T-Cell Lymphoma

**DOI:** 10.7759/cureus.71757

**Published:** 2024-10-18

**Authors:** Soraia G Araújo, Martinha M Vale, Inês M Araújo, Raquel Azevedo, Ana Oliveira

**Affiliations:** 1 Critical Care Medicine, Hospital de Braga, Braga, PRT; 2 Internal Medicine, Hospital de Braga, Braga, PRT; 3 Infectious Disease, Hospital de Braga, Braga, PRT

**Keywords:** adenopathies, bone lytic lesions, lymphomas, peripheral t-cell lymphoma angioimmunoblastic, peripheral t-cell lymphoma not otherwise specified

## Abstract

Peripheral T-cell lymphoma (PTCL) is a heterogeneous group of uncommon and frequently severe lymphomas. There are around 30 different subtypes, with peripheral T-cell lymphoma, not otherwise specified (PTCL-NOS) and angioimmunoblastic T-cell lymphoma having the poorest outcomes under standard chemotherapy. We discuss two cases with unusual initial presentations of these aggressive subtypes of peripheral T-cell lymphoma.

The first case is a peripheral T-cell lymphoma, not otherwise specified, which initially presents with pain in the left hip. For six months, this pain was erroneously labeled as degenerative osteoarticular pathology. When exploring further the patient's symptoms, one can see a set of constitutional symptoms that accompany the pain and end up sounding alarm bells. Imaging examinations reveal a lytic bone lesion in the iliac crest and multiple adenopathy formations. The bone lesion biopsy provides a definitive diagnosis, and after six cycles of chemotherapy, the control examinations reveal a complete remission.

The second case is a nodal T-follicular helper lymphoma, angioimmunoblastic subtype, that initially appeared to be a respiratory infection that did not respond to antibiotic therapy. However, by carefully reviewing the anamnesis, it was possible to identify constitutional symptoms that should have alerted to a different diagnosis. Imaging examinations confirmed the suspicion and reported numerous adenopathies as well as splenomegaly associated with splenic infarction. Axillary adenopathy biopsy dictated the diagnosis. The patient completed six cycles of chemotherapy with a complete response.

These cases demonstrate how crucial it is to keep an elevated level of suspicion for this uncommon pathology to avoid delays in diagnosis and thereby maximize the chance of a successful outcome.

## Introduction

Peripheral T-cell lymphoma (PTCL) is a heterogeneous group of quite rare and often aggressive lymphomas. It accounts for approximately 15% of all cases of non-Hodgkin lymphoma (NHL) [1,2]. There are over 30 different subtypes of PTCL, and the borders between the various subtypes are still blurred [2].

PTCL, not otherwise specified (PTCL-NOS) is the most common subtype and is a highly heterogeneous group of mature post-thymic T-cell tumors, which does not fit into any other well-defined subtype [2,3]. The mean age of diagnosis is around 60 years of age and is more prevalent in males [4]. In most cases, it presents with widespread nodal disease, but it can also have extranodal involvement, especially in the skin and gastrointestinal tract [4]. Angioimmunoblastic T-cell lymphoma is the second most common subtype of peripheral T-cell lymphoma and is responsible for 2% of all NHL [1]. In the recent fifth edition (2022) of the WHO Classification of Haematolymphoid Tumours, this subtype changed its denomination and is now named nodal T-follicular helper lymphoma, angioimmunoblastic (TFH-AITL) subtype [2]. The TFH-AITL subtype is characterized by diffuse lymphadenopathy and a tendency to autoimmune events [2].

Among all subtypes, PTCL-NOS and TFH-AITL subtypes are those that present the worst outcome under standard chemotherapy [2]. We present two cases with atypical initial presentation of PTCL-NOS and TFH-AITL subtypes.

## Case presentation

Case 1

A 71-year-old Caucasian female presented to the hospital due to pain in the left hip that limited walking. It has been ongoing for six months and progressively worsening. During this period, she visited two urgent care clinics and was treated with analgesics for likely osteoarticular pathology. She had no relevant medical history. When carrying out a more detailed anamnesis, she reported complaints of anorexia, asthenia, and involuntary weight loss (14.5% of her weight in one year), associated with night sweats and generalized itching.

Her vital signs revealed a blood pressure of 148/73 mmHg, heart rate of 87/minute, sinus rhythm, respiratory rate of 17/minute, temperature of 96.8°F, and oxygenation saturation of 96% on room air. Objective examination revealed a soft, painful mass on the left iliac crest and palpable bilateral axillary lymphadenopathy (measuring approximately 10 mm, mobile and non-painful).

**Figure 1:**
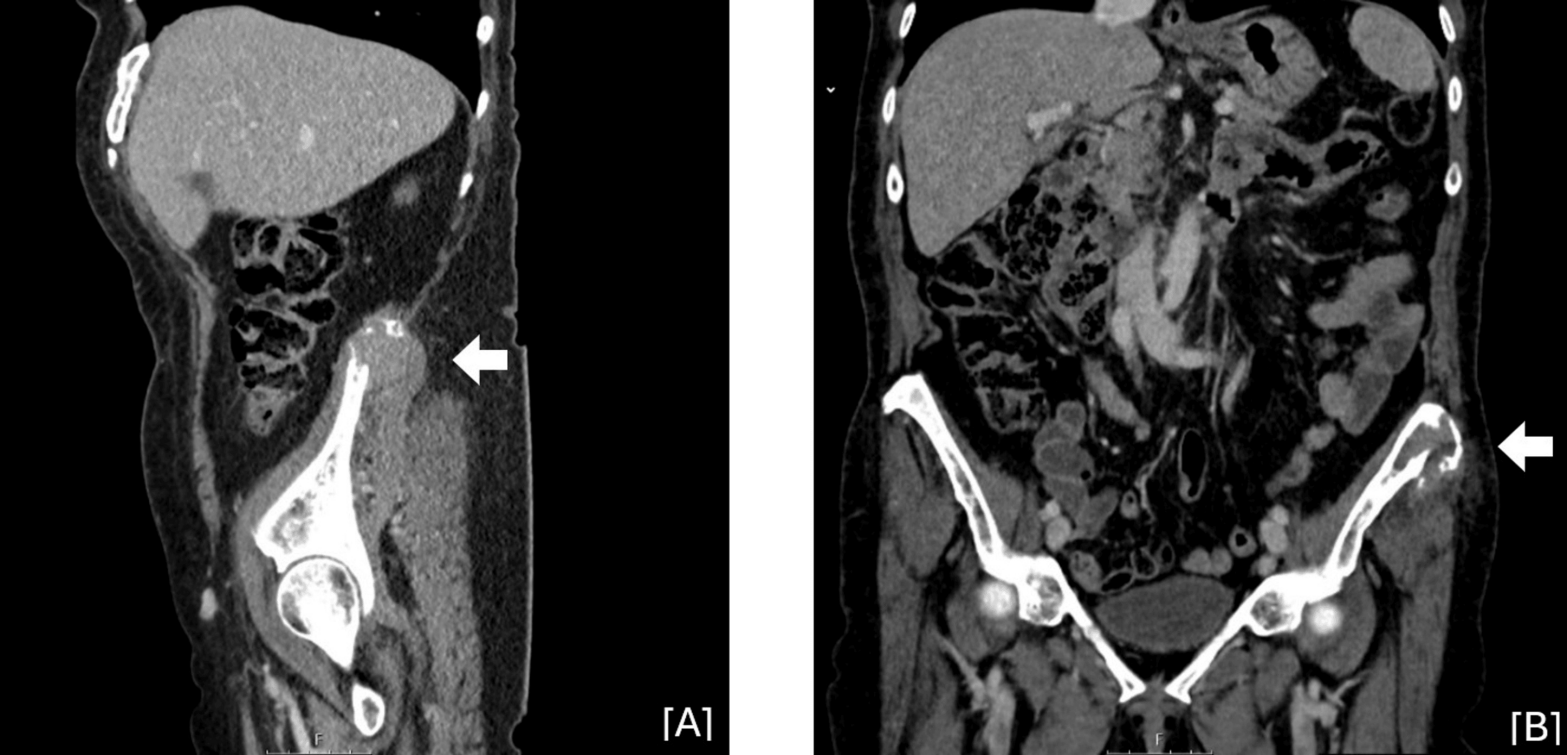
Abdominal and Pelvic Computed Tomography A large bone lesion with a soft tissue component on the right iliac wing (A, white arrow) measuring approximately 56 mm and another lesion on the left anterior superior iliac spine (B, white arrow) measuring approximately 32 mm

**Figure 2:**
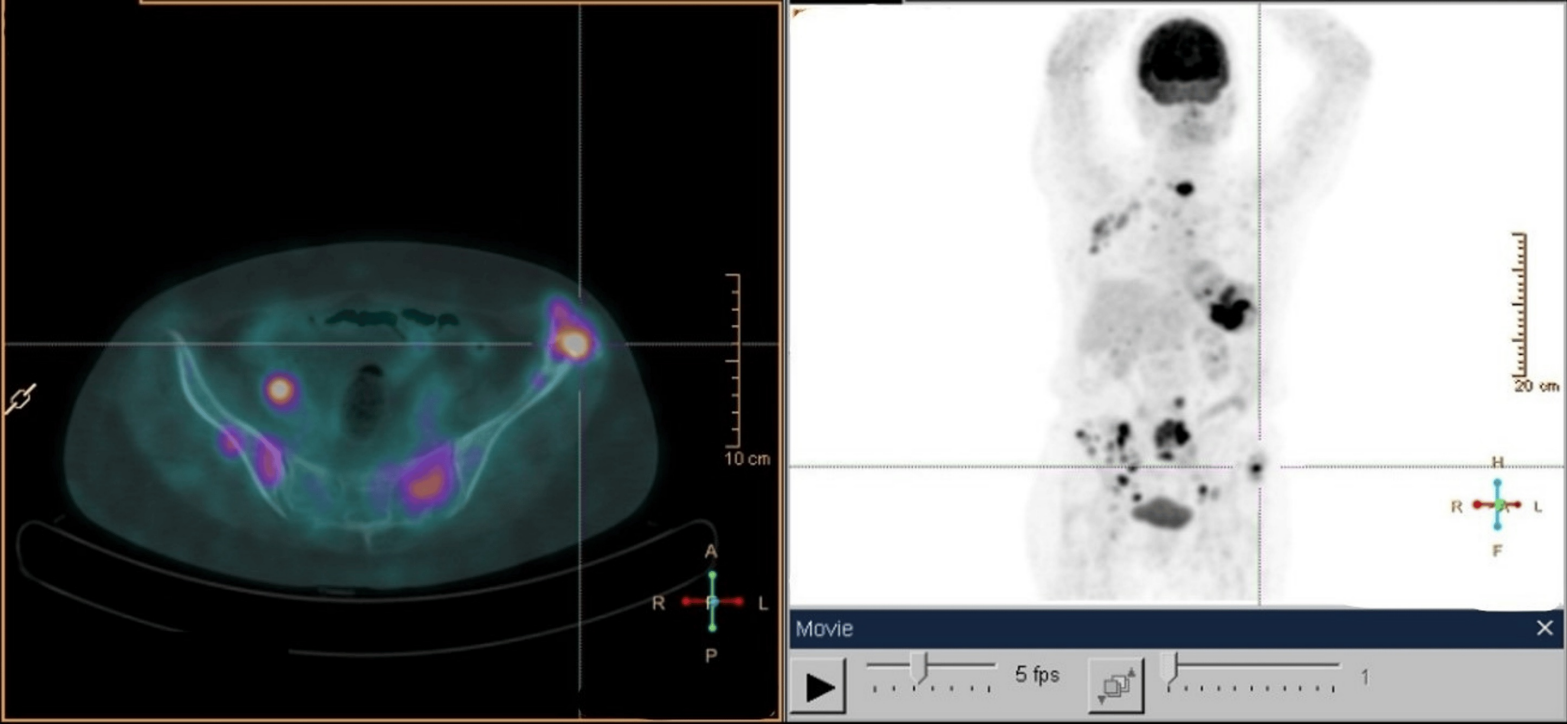
18F-FDG PET-CT PET-CT suggesting malignant neoplastic involvement in the supra- and infra-diaphragmatic lymph nodes, splenic, and bone (multiple lesions involving pelvic bones, multiple vertebral elements, the sternum, and the right seventh costal arch). with possible involvement of the gastric fundus 18F-FDG PET-CT: 18F-fluorodeoxyglucose positron emission tomography-computed tomography

Laboratory studies only identified mild lymphocytosis (3,600/uL) (normal range: 1,200-3,400/uL) and increased alkaline phosphatase (141 U/L) (normal range: 46-116 U/L), lactate dehydrogenase (1,060 U/L) (normal range: 120-246 U/L), and beta-2 microglobulin (3,585 ng/mL) (normal range: 1,000-2,400 ng/mL). An X-ray, ultrasound, and computed tomography (CT) detected two large bone lytic lesions, one on the left iliac crest and the other on the right iliac wing, involving soft tissues, reflecting probable secondary lesions (Figure 1). CT scans with contrast of the chest, abdomen, and pelvis (CAP) identified bilateral axillary adenopathies, the largest on the right measuring 30 mm, as well as several ganglionic formations in the lumbar aortics and root of the mesentery (Figure 1). Positron emission tomography (PET) suggests supra- and infra-diaphragmatic, splenic, and multifocal bone neoplastic involvement, as well as possible gastric involvement (Figure 2).

A biopsy of the lytic lesion and axillary node was performed, which demonstrated a proliferation of lymphocytes suggestive of lymphoproliferative disease of the non-Hodgkin lymphoma type. The immunohistochemical study of the bone lesion confirms the final diagnosis of peripheral T-cell lymphoma, non-specific subtype, stage IVB (bone involvement). The patient underwent chemotherapy with cyclophosphamide, doxorubicin, vincristine, and prednisone (CHOP) regimen, with good tolerance. A reevaluation PET scan was performed after six cycles, which revealed a complete metabolic response.

Case 2

An 83-year-old Caucasian male presented to the hospital complaining of fever, sweating, cough, and myalgia that had lasted two weeks. About 10 days before, he was seen by a doctor and diagnosed with community-acquired pneumonia, for which he completed a course of antibiotics. Due to the lack of improvement, he returned to the hospital. When taking a more detailed anamnesis, the patient reported important constitutional symptoms that were initially undervalued, such as anorexia, asthenia, sweating, and signs of abdominal swelling lasting three weeks. He also noticed involuntary weight loss of 6% of his body weight in three months. According to his personal history, the patient had hypertension and dyslipidemia.

His vital signs revealed a blood pressure of 131/81 mmHg, heart rate of 83/minute, sinus rhythm, respiratory rate of 14/minute, temperature of 97.9°F, and oxygenation saturation of 98% on room air. Objective examination revealed hepatosplenomegaly and palpable bilateral axillary and submandibular lymphadenopathy (measuring approximately 20 mm, mobile and non-painful).

**Figure 3:**
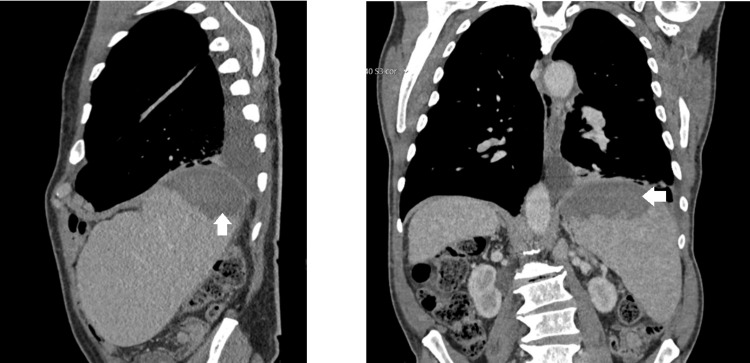
Thoracic and Upper Abdominal Computed Tomography Enlarged spleen measuring approximately 18 cm in longitudinal diameter and presenting a hypodense area at the upper pole measuring approximately 84 mm in transverse diameter, compatible with splenic infarction (white arrows)

**Figure 4:**
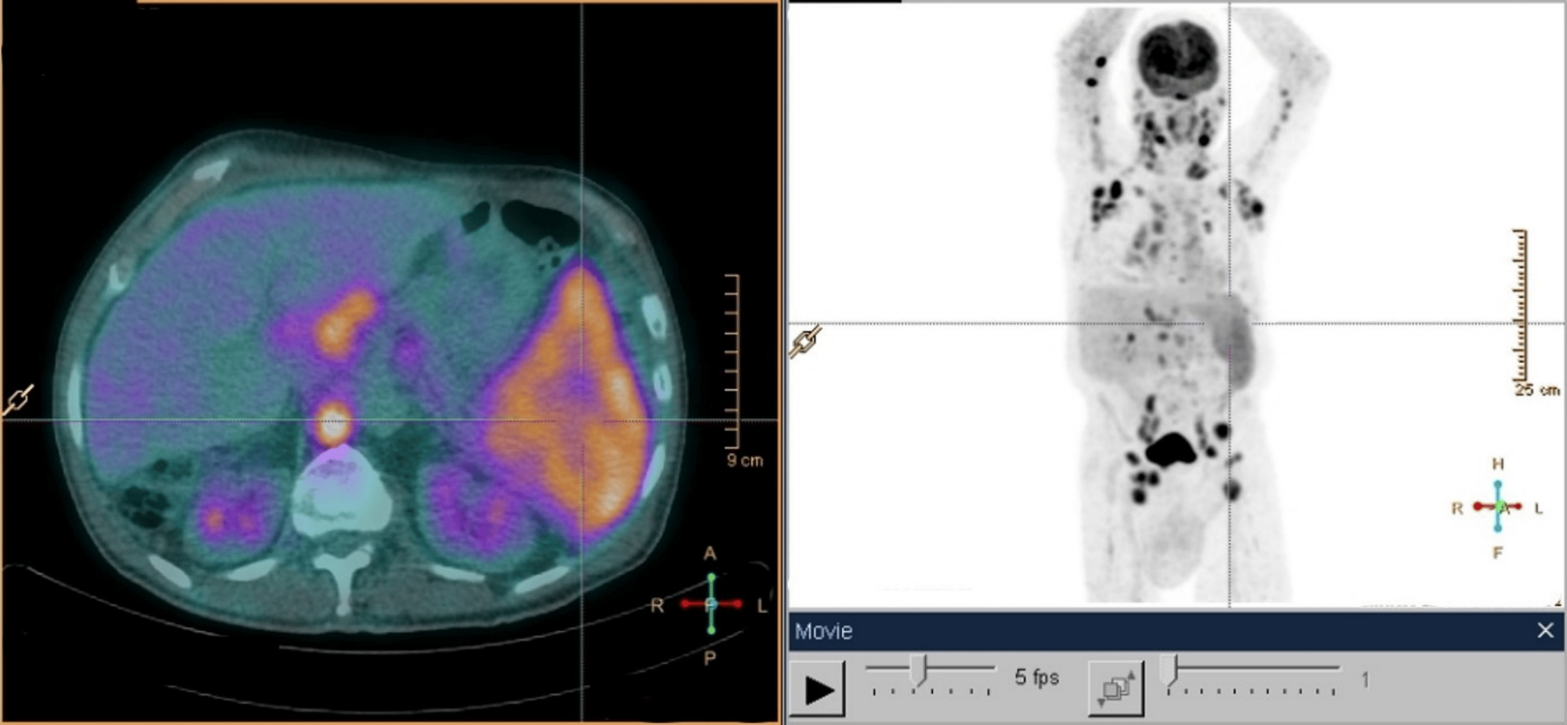
18F-FDG PET-CT PET-CT suggesting supra- and infra-diaphragmatic nodal, nasopharyngeal, and splenic involvement 18F-FDG PET-CT: 18F-fluorodeoxyglucose positron emission tomography-computed tomography

Laboratory studies only identified an increase in alkaline phosphatase (159 U/L) (normal range: 46-116 U/L), lactate dehydrogenase (480 U/L) (normal range: 120-246 U/L), beta-2 microglobulin (9,490 ng/mL) (normal range: 1,000-2,400 ng/mL), and immunoglobulin A (1,422 mg/dL) (normal range: 40-350 mg/dL). A CT of the CAP detected multiple pericentimetric adenopathies in all mediastinal compartments, several bilateral axillary adenopathies, and multiple retroperitoneal pericentimetric adenopathies. A moderate pleural effusion on the left and an enlarged spleen with a hypodense area in the upper pole compatible with splenic infarction were also revealed (Figure 3). PET suggests countless hypermetabolic, supra- and infra-diaphragmatic adenopathies, and splenic involvement, as well as involvement of the posterior portion of the nasopharynx (Figure 4).

A biopsy of an axillary node was performed, which showed a proliferation of medium and large lymphoid cells, with clear cytoplasm and large nuclei, some lobulated and with an evident nucleolus, immunoreactive for CD2, CD3, CD4, and CD5 and negative for CD20, PAX-5, CD7, CD8, CD10, CD30, and ALK. The morphological aspects described and the immunohistochemical profile, despite CD10 negativity, were very suggestive of angioimmunoblastic T-cell lymphoma. For staging, a bone marrow biopsy was performed, which excluded involvement by lymphoma. The final diagnosis was nodal T-follicular helper lymphoma, angioimmunoblastic subtype, stage IIIB. The patient completed treatment with six cycles of chemotherapy with the CHOP regimen, with good tolerance. A reevaluation PET scan was performed after four cycles, which revealed a complete metabolic response.

## Discussion

These cases portray two subtypes of rare PTCL, with challenging and unclear initial presentations requiring detailed attention from the clinician. The initial symptoms are not always obvious, and sometimes, an incomplete anamnesis delays the diagnosis.

On the other hand, a patient complaining of hip pain, as in case 1, would have a multitude of diagnoses that are more likely than lymphoproliferative disease, including joint degenerative pathology, inflammatory pathology (arthritis, bursitis, and tendinitis), nerve compression, or bone fracture. However, none of this justified weight loss, asthenia, or anorexia. Constitutional symptoms indicated a possible neoplastic etiology. Of all the tumors associated with lytic bone lesions, lymphoma was the most probable considering the adenopathies, itching, and night sweats. The same logic applies to case 2, which centers around a patient with a cough, myalgia, fever, and sweating. The most likely differential diagnosis in this scenario is bacterial or viral respiratory infection. Knowing that the patient also had asthenia, anorexia, weight loss, and adenopathies, other conditions must be ruled out, such as tuberculosis, sarcoidosis, and carcinomas. The signs of hepatosplenomegaly could raise the suspicion of lymphoproliferative disease, but a histological test is always needed to establish the diagnosis.

Both cases left some key clues for the diagnosis that should be highlighted. First, T-cell lymphomas are typically diagnosed at older ages, with a median incidence between 65 and 70 years [1]. In both cases, patients are within the age group with the highest incidence of lymphomas. Another important clue was the presence of B symptoms. After carrying out detailed anamnesis, in both cases, there was a clear presence of constitutional symptoms that had already evolved for some time and were initially devalued. The same applies to the presence of adenopathies. Both patients had adenopathies that were easily accessible and palpable on physical examination, so whenever a patient presents constitutional symptoms, it is essential to search for adenopathies. In these two cases, the clinician simply needed to carry out a more careful physical examination to find what is the most alarming clue of lymphoma.

Furthermore, our knowledge about T-cell lymphomas, compared to what we already know about B lymphomas, is still very rudimentary [5], which contributes to the diagnosis being made in more advanced stages of the disease. Adding the fact that among all subtypes of PTCL, PTCL-NOS and TFH-AITL are the ones with the worst prognosis under conventional chemotherapy [2] makes the chances of remission even lower.

These two specific cases are two good but infrequent examples of a positive response to therapy in these subtypes of PTCL. In fact, with this case report, we pretend to emphasize the importance of collecting a complete anamnesis, carrying out a careful physical examination, and keeping a high level of suspicion to detect these lymphoproliferative diseases early. The ultimate objective is always to contribute to a better outcome and, in the best-case scenario, a complete remission of the disease.

## Conclusions

These cases illustrate two distinct types of uncommon PTCL, with atypical initial presentations that required the clinician's consideration. Initially presenting with left hip pain, the first case was PTCL-NOS. Further investigation of the patient's symptoms revealed a set of constitutional symptoms, palpable adenopathies, itching, and night sweats. Imaging tests showed numerous adenopathy forms and a lytic bone lesion in the iliac crest. A definitive diagnosis was made by the bone lesion biopsy, and following six rounds of chemotherapy, the control examinations showed total remission. The second case was a TFH-AITL, which initially seemed to be a respiratory tract infection. However, the patient also presented anorexia, asthenia, and weight loss. Imaging tests revealed multiple adenopathies and splenomegaly associated with splenic infarction. A biopsy of the axillary adenopathy confirmed the diagnosis, and after six cycles of chemotherapy, the patient had a complete metabolic response.

Ultimately, we want to highlight the importance of maintaining a high level of suspicion for these rare lymphomas to prevent delays in diagnosis and initiation of treatment, thus affecting the vital prognosis. These two cases serve as excellent illustrations of how prompt treatment can be beneficial for these aggressive PTCL subtypes, highlighting the importance of maintaining an elevated level of suspicion for this rare pathology to prevent delays in diagnosis. The goal is to contribute to a better outcome and, in the most ideal scenario, complete illness remission.
